# GHOST: global hepatitis outbreak and surveillance technology

**DOI:** 10.1186/s12864-017-4268-3

**Published:** 2017-12-06

**Authors:** Atkinson G. Longmire, Seth Sims, Inna Rytsareva, David S. Campo, Pavel Skums, Zoya Dimitrova, Sumathi Ramachandran, Magdalena Medrzycki, Hong Thai, Lilia Ganova-Raeva, Yulin Lin, Lili T. Punkova, Amanda Sue, Massimo Mirabito, Silver Wang, Robin Tracy, Victor Bolet, Thom Sukalac, Chris Lynberg, Yury Khudyakov

**Affiliations:** 1 Molecular Epidemiology and Bioinformatics Laboratory, Division of Viral Hepatitis, Centers for Disease Control and Prevention, Atlanta, USA; 2 Department of Computer Science, Georgia State University, Atlanta, USA; 3 NCHHSTP Informatics Office, Centers for Disease Control and Prevention, Atlanta, USA; 4 IT Research and Development Office, Centers for Disease Control and Prevention, Atlanta, USA; 5 Centers for Disease Control and Prevention, ITSO Application Hosting Branch, Atlanta, USA; 6 Northrop Grumman Corporation, Falls Church, USA

**Keywords:** HVR1, HCV, Liver cancer, Threshold, Transmission, Outbreak detection, Surveillance, Public health, Cloud, Virtual diagnostics

## Abstract

**Background:**

Hepatitis C is a major public health problem in the United States and worldwide. Outbreaks of hepatitis C virus (HCV) infections associated with unsafe injection practices, drug diversion, and other exposures to blood are difficult to detect and investigate. Effective HCV outbreak investigation requires comprehensive surveillance and robust case investigation. We previously developed and validated a methodology for the rapid and cost-effective identification of HCV transmission clusters. Global Hepatitis Outbreak and Surveillance Technology (GHOST) is a cloud-based system enabling users, regardless of computational expertise, to analyze and visualize transmission clusters in an independent, accurate and reproducible way.

**Results:**

We present and explore performance of several GHOST implemented algorithms using next-generation sequencing data experimentally obtained from hypervariable region 1 of genetically related and unrelated HCV strains. GHOST processes data from an entire MiSeq run in approximately 3 h. A panel of seven specimens was used for preparation of six repeats of MiSeq libraries. Testing sequence data from these libraries by GHOST showed a consistent transmission linkage detection, testifying to high reproducibility of the system. Lack of linkage among genetically unrelated HCV strains and constant detection of genetic linkage between HCV strains from known transmission pairs and from follow-up specimens at different levels of MiSeq-read sampling indicate high specificity and sensitivity of GHOST in accurate detection of HCV transmission.

**Conclusions:**

GHOST enables automatic extraction of timely and relevant public health information suitable for guiding effective intervention measures. It is designed as a virtual diagnostic system intended for use in molecular surveillance and outbreak investigations rather than in research. The system produces accurate and reproducible information on HCV transmission clusters for all users, irrespective of their level of bioinformatics expertise. Improvement in molecular detection capacity will contribute to increasing the rate of transmission detection, thus providing opportunity for rapid, accurate and effective response to outbreaks of hepatitis C. Although GHOST was originally developed for hepatitis C surveillance, its modular structure is readily applicable to other infectious diseases. Worldwide availability of GHOST for the detection of HCV transmissions will foster deeper involvement of public health researchers and practitioners in hepatitis C outbreak investigation.

## Background

Worldwide, almost 3% of people are infected with hepatitis C virus (HCV) [1]. Approximately 80% of HCV infections develop into the chronic state [2]. Of these, 15–30% will be diagnosed with liver fibrosis or cirrhosis, and 5% will die from cirrhosis or hepatocellular carcinoma (HCC) [3]. Globally, liver cancer is the second most common form of cancer death [2], and in the United States, occurrences are increasing at a higher rate than any other form of cancer except thyroid cancer [4]. An estimated 2.7–3.9 million Americans live with HCV infection [5]. In the United States, in 2007, the number of deaths related to HCV overtook the number of human immunodeficiency virus (HIV)-related deaths [6]. In 2012, 22,972 Americans died of liver cancer [7], 28,972 were newly diagnosed [7], and HCV-related deaths surpassed all 60 other nationally notifiable diseases combined [8].

Like HIV, HCV is primarily transmitted through parenteral exposures. In the 1960s, 1970s, and 1980s, before the virus was discovered and HCV screening of the blood supply was standard practice, HCV infection was expanding worldwide. In the general US population, HCV infection is particularly high among individuals born between 1945 and 1965 [9–11]. However, there has been a 151% increase in reported HCV infections in the United States between 2010 and 2013 and typically in non-urban areas, corresponding to the surge in opioid addiction and injection drug use (IDU) among individuals born after 1986 [12]. HCV is the most common infection with a transmission path through IDU which accounts for the most significant proportion of newly acquired HCV infections [13–17]. During a recent HIV outbreak investigation in Indiana, among the 181 initial HIV infected individuals identified, 92% were found to be coinfected with HCV [18]. Successful HCV surveillance programs are fundamental for the implementation of public health interventions aimed at interrupting HCV transmission.

HCV exists as a population of numerous variants in each infected individual [19]. It has been observed that minority variants in the source are often those responsible for transmission [20, 21], a situation that precludes the use of a single sequence per individual because many such transmissions would be missed [22]. Computational analysis of the NGS data for the detection of HCV transmission is a very complex process and requires significant expertise in application of phylogenetic methods and interpretation of phylogenetic data within an epidemiological context.

We previously developed and validated a methodology for the rapid, accurate, and cost-effective identification of transmission clusters using large samples of intra-host HCV variants obtained by next-generation sequencing (NGS) using a genetic distance threshold derived with EPLD PCR data and validated on NGS 454Jr data. When applied to the Hypervariable Region 1 (HVR1), the method discriminated clusters of related samples from unrelated samples with 100% sensitivity and 100% specificity [23]. Calculating the distances between all sequences in a set of samples is an extremely computationally demanding task, and so we have also evaluated a set of filters that can eliminate sample pair comparisons and greatly reduce the computational cost [24]. The Hamming radius filter was found to perform best individually, accurately filtering up to 91% of all pairwise sequence comparisons from consideration [24]. Here, we validate this threshold against the Illumina MiSeq platform employing a modified Hamming radius filter, a cloud-based distributed infrastructure, and other computational techniques to accommodate the scale and characteristics of MiSeq data.

We introduce Global Hepatitis Outbreak and Surveillance Technology (GHOST) - a cloud-based system that is composed of a set of bioinformatics tools for controlling quality of NGS data and automatic extraction of information on transmission clusters. GHOST integrates bioinformatics and information technologies and enables all users, regardless of their computational expertise, to conduct independent, accurate, and reproducible HCV molecular surveillance. We have adapted GHOST to the Illumina platform. Here, we describe several implemented algorithms and explore performance of the system in analysis of known genetically related and unrelated HCV strains. Relevant public health information is automatically obtained by GHOST from HCV genetic data in a form suitable for guiding effective intervention measures. Access to GHOST is available to all authenticated users for conducting accurate outbreak investigations and molecular surveillance.

### Implementation

#### Sequencing platform

The original transmission detection algorithms [24] were designed for the 454Jr platform and have now been adapted to the Illumina platform, particularly MiSeq. The MiSeq’s deeper sequencing capability provides a more comprehensive snapshot of the population spectrum per sample and a cost-effective way to gain greater sample-pooling per run. Whereas the 454 error correction method was concerned with substitutions, insertions, and deletions, including those associated with homopolymer errors, GHOST’S MiSeq error correction focuses on substitution.

#### GHOST hepatitis C virus (HCV) sequencing protocol

The GHOST HCV sequencing protocol uses a novel amplicon-based sequencing method that targets the HVR1 of the HCV genome. This region was chosen for its relatively high variability, allowing for fine-grained assessment of evolutionary distances arising from recent transmission events. In order to sequence several samples per run and reduce costs, it utilizes a hierarchical multiplexing scheme with an additional pair of identifiers that persist after standard Illumina demultiplexing to minimize intra-run mis-assignments. GHOST processes Illumina MiSeq 300 bp paired-end reads with full overlap of the forward and reverse reads to redundantly sequence each amplicon, and the redundant information is used to reduce sequencing errors. A detailed HCV MiSeq sequencing protocol is available on the GHOST website to authenticated users or upon request.

#### GHOST web interface

**Fig. 1:**
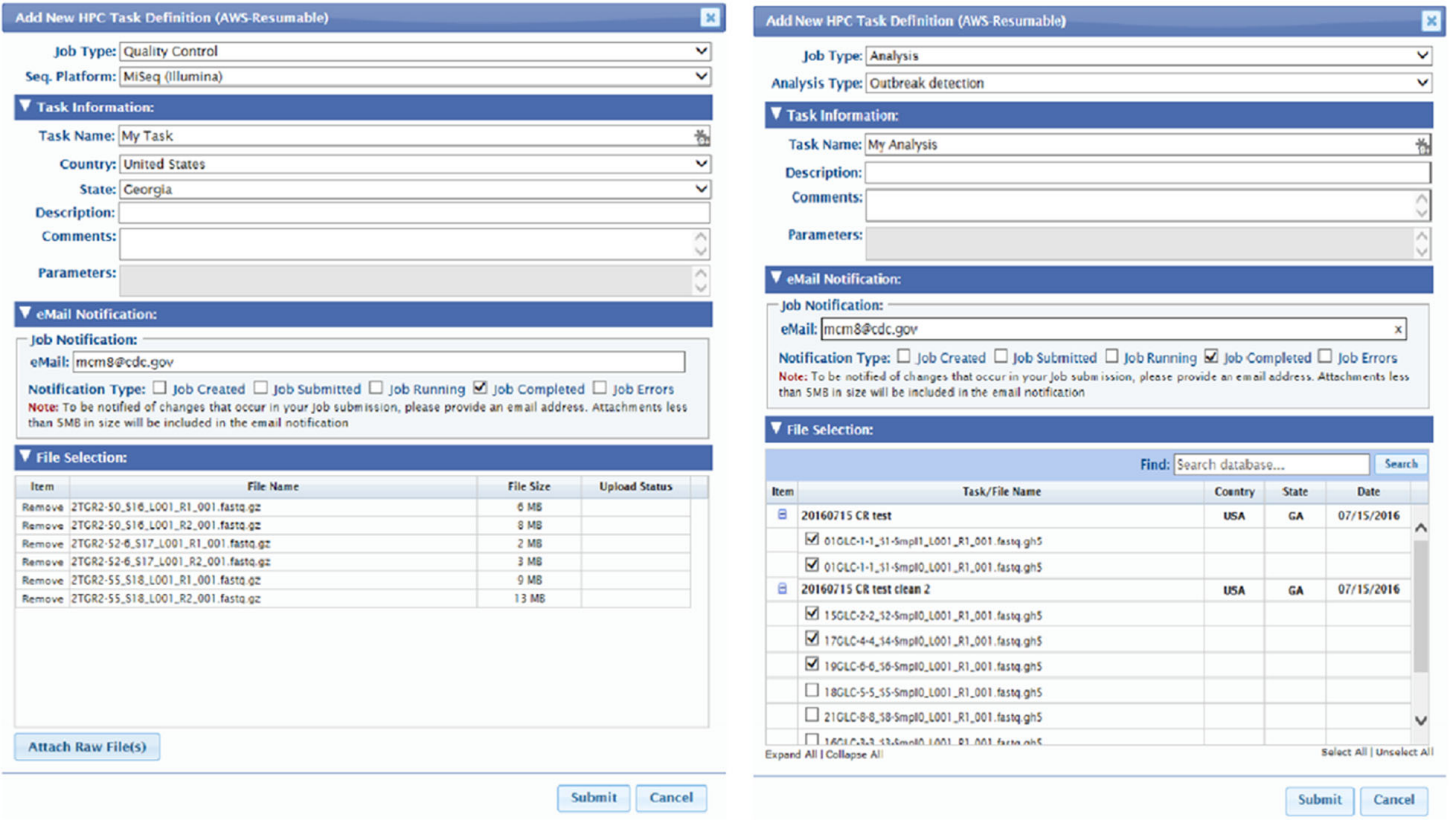
Screen captures of the two main types of tasks within the GHOST web interface. Left shows the Quality Control task. Right shows the Analysis task

GHOST users are typically local public health researchers, outbreak investigators and those involved in molecular surveillance. The first step for GHOST users is to submit a user account request. The GHOST administrators authenticate and validate the user request and to ensure the user will have secure access to the system. After access is granted, the user can choose either of two main web-based tasks: Quality Control or Analysis (Fig. 1). Each workflow is described below.

#### GHOST quality control tasks

**Fig. 2:**
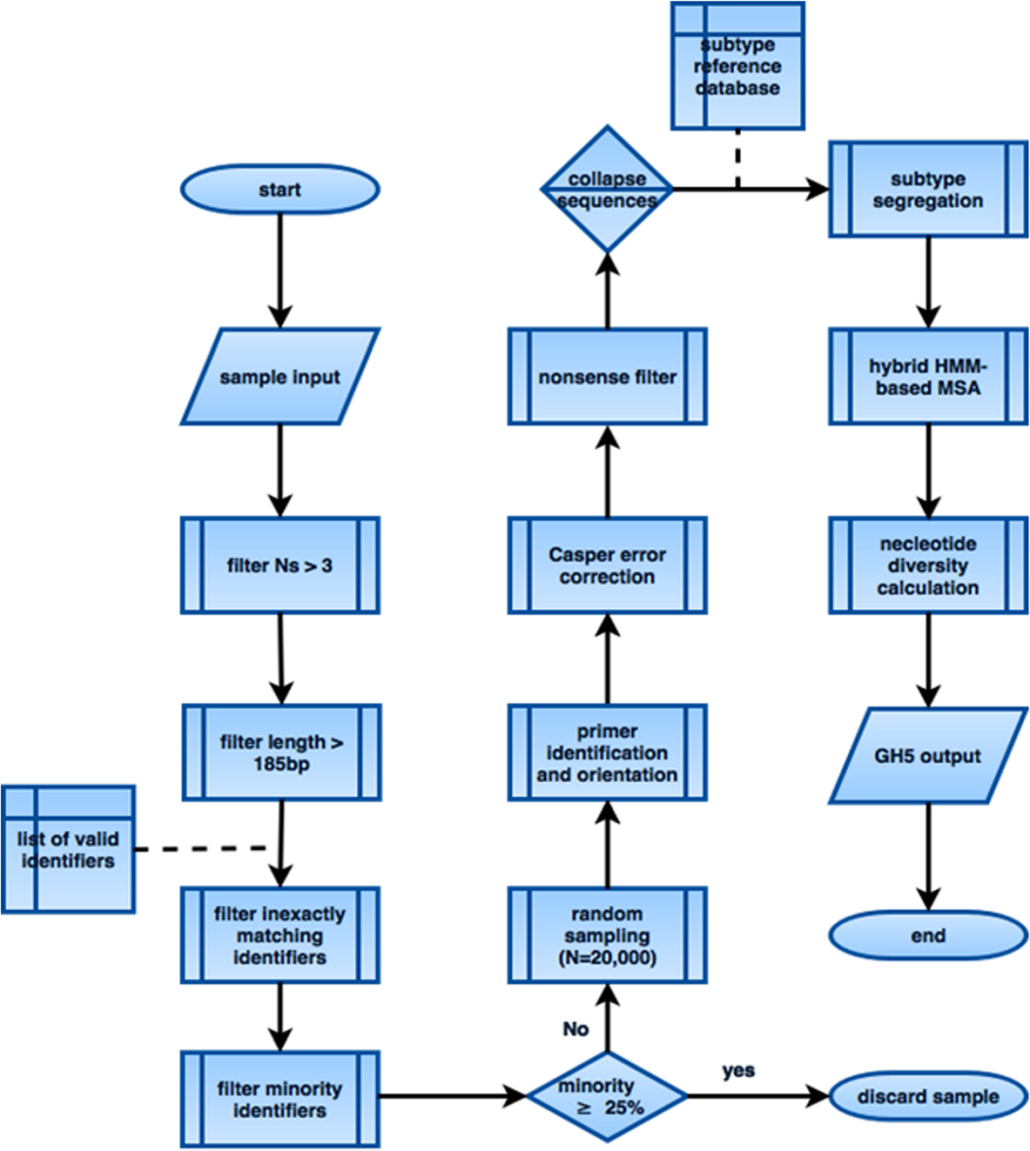
Flowchart depicting processing steps for a Quality Control task

The Quality Control (QC) task is designed for the upload of sequence data directly after Illumina sequencing and demultiplexing. It takes gzip-compressed fastq formatted data and expects filenames in accordance with the conventional Illumina-named gzip-compressed fastq naming. After standard demultiplexing*,* read pairs are filtered out if a read has more than three N’s or has a length less than 185 bp. Each identifier on both forward and reverse reads are examined and the pair is discarded if either identifier is found to not be an exact match to a given list of valid identifiers. Pairs containing valid identifiers are discarded if they are not a constituent of the majority identifier tuple. If 25% or more of the read pairs are found to contain valid identifiers that are not the majority tuple, the entire sample is discarded from analysis without further processing. Owing to computational limitations, a random subsample of *N* = 20,000 read pairs are taken by the unweighted reservoir sampling method [25, 26] and searched for the forward and reverse reads. Primer sequences are located in each read using fuzzy matching and only allow substitutions ≤2, insertions (relative to the reference) ≤ 1, deletions (relative to the reference) ≤ 1, and a combination of total errors ≤3. Read pairs where either primer cannot be found are discarded. The primer locations are used to orient the reads into the uniform orientation. Read pairs are unified into a single error-corrected sequence using the Casper error correction method [27] with a quality threshold of 15, *k*-mer length of 17, *k*-mer neighborhood of 8, and minimum match threshold of 95%. Overlap fitness is evaluated by the classical Hamming Distance. The overlap corresponding to the highest ratio of correct positions to overlap length is selected, with the longest overlap being preferred in the event of there being more than one overlap with equal ratios. Merged sequences are discarded if a nonsense-free reading frame cannot be found. Those not discarded are collapsed into unique occurrences with associated frequencies, thereby reducing subsequent computation time and associated cost. Sequences are then segregated into subtypes using the blastn program included in blast + toolkit v2.3.0 [28] with an in-house curated reference database and the following adjusted parameters: minimum E-value 30.0, word size 7, gap opening penalty 2, and a minimum raw gapped score 95. The total normalized bit score of each high-scoring segment is calculated with respect to the genotype and subtype of each reference sequence. The log probability of observing the bit score larger than this is calculated using Eq. 5 in Karlin and Altschul [29], and the best match is used to classify the sequence into a subtype category. Any sequences whose best score is less than the log probability of −135 is discarded as non-HCV. The sequences are aligned using a hybrid strategy of traditional multiple sequence alignment using MAFFT v7.215 [30] for the most frequently occurring 1000 sequence variations, and the resultant alignment is used to create a Hidden Markov Model (HMM) seed for subsequent HMM-based alignment of the remaining sequences using HMMer v3.1b1 [31]. The consensus, nucleotide diversity, and largest Levenshtein distance from the consensus (radius) are calculated per subtype present in the sample. The QC task then writes preprocessed files in the in-house HD5-derived GH5 format containing the haplotypes found with associated frequencies and other metadata (Fig. 2).

#### GHOST analysis tasks

The Analysis task uses as input a user-defined set of cleaned GH5 files that result from the QC task workflow. Currently, the analysis task has a single module for the detection of HCV transmissions, where genetic distances between all sample pairs are measured to determine if any fall below the experimentally validated distance threshold [23]. The use of Ultra-Deep Sequencing (UDS) data immensely increases the sensitivity of transmission detection but brings a considerable computational challenge: calculating the minimum distance between all samples. Several techniques were employed to minimize both runtime and memory usage, the four main ones being: (i) random subsampling of the original file as aforementioned, (ii) a variation of the Hamming radius filter of sample-pair candidates we termed the metric filter [24], (iii) HMM-based multiple sequence alignment (MSA), and (iv) optimized distance calculation [24].

In *Rytsareva* et al. [24], it was found that for two single-subtype samples S_1_ and S_2_, with consensuses C_1_ and C_2_ and Hamming radii R_1_ and R_2_, then the samples cannot have a sequence pair with distance lower than the threshold (T) if dist(C_1_, C_2_) – (R_1_ + R_2_) > LT, where L is the length of the sequence alignment. We made two modifications to this filter: (i) We implemented a variation of this filter employing a modified Hamming distance we termed “corrected Hamming distance” that does not count positions with insertions or deletions as differences, and (ii) the alignment-independent Levenshtein distance was used for radii calculation.

For each subtype in each sample, the consensus and radius produced in the preprocessing step are used to establish the metric filter parameters, and groups are removed from the candidate list accordingly. This filter significantly reduces the proportion of full distance calculations performed and greatly reduces the computational cost without any loss of information. For group pairs not removed by the metric filter, alignments for the remaining distance calculations use the same HMM method described above. Corrected Hamming distance calculations are performed with an optimized distance calculator named HDIST – an in-house algorithm optimized to minimize pipeline stalls and maximize cache usage by converting sequence pairs into groups of non-overlapping 3-mers, then to base-5 integers that are used as indices in a pre-calculated look-up table. The choice of the k-mer was empirically tested using a range of k-mers and the choice of 3-mers was found to be the size maximizing cache memory hits. Sequence pairs whose distance is below the threshold are not considered if either sequence has a frequency of one. The Analysis task outputs an intuitive transmission network graph. Nodes represent input samples, and edges connect sample pairs found to have subpopulations with a distance below the threshold.

#### The computational platform

**Fig. 3:**
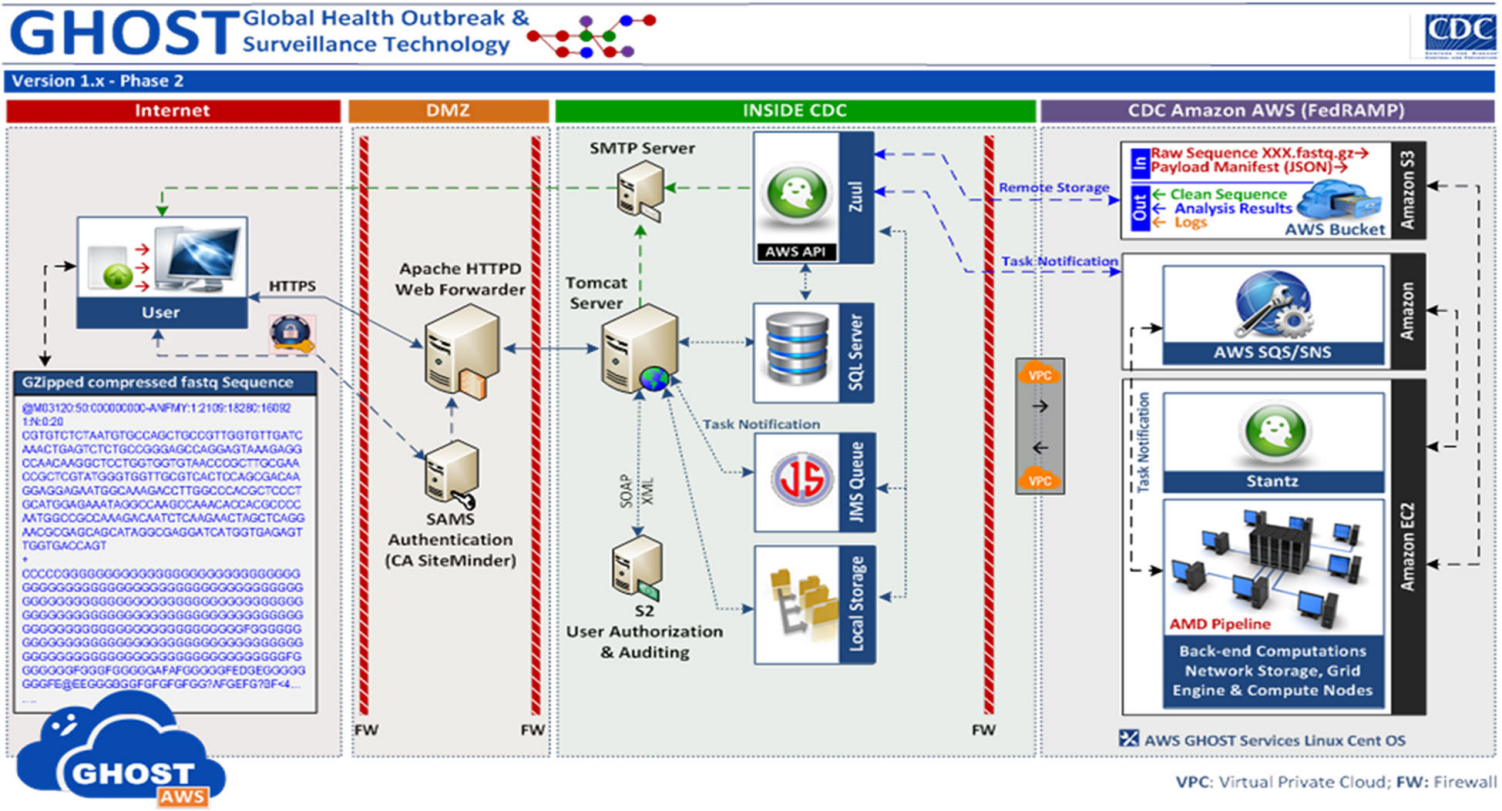
The technological layout of the GHOST system. From left to right: user uploading Illumina demultiplexed sequence data; front-end public exposure, authentication, and message forwarding; middle-tier control, messaging, and data management within the CDC, backend computation and control management within the AWS environment

The GHOST back-end and HCV transmission analyses are implemented using a combination of Python, Cython, and command line programs. Python libraries include numpy/scipy for general computational support, biopython for sequence manipulation, regex for fuzzy regular expression matching, h5py for data storage, networkx for storage and processing of transmission networks. Back-end execution is performed via the Amazon Web Services (AWS) using AWS Simple Storage Service (S3) for storage and the Amazon Elastic Compute Cloud (EC2) with 26 configured nodes with two acting as management nodes and 24 acting as compute nodes. The front-end and middle-tier are a composite of technologies including HTML, D3, Javascript, Java, JSON, and XML. A set of services were developed to standardize communication from the front-end to the AWS platform: (i) the “Zuul” service is responsible for moving data into and out of S3 and communicating with AWS components using the AWS SQS API. Zuul also provides the status of task execution processes back to users. (ii) The “Stantz” service acts as a control point within the EC2 platform communicating with Zuul and performing cluster management and oversight functions using Open Grid Engine and the Distributed Resource Management Application API (DRMAA) (Fig. 3).

## Results

### Benchmarking

**Fig. 4:**
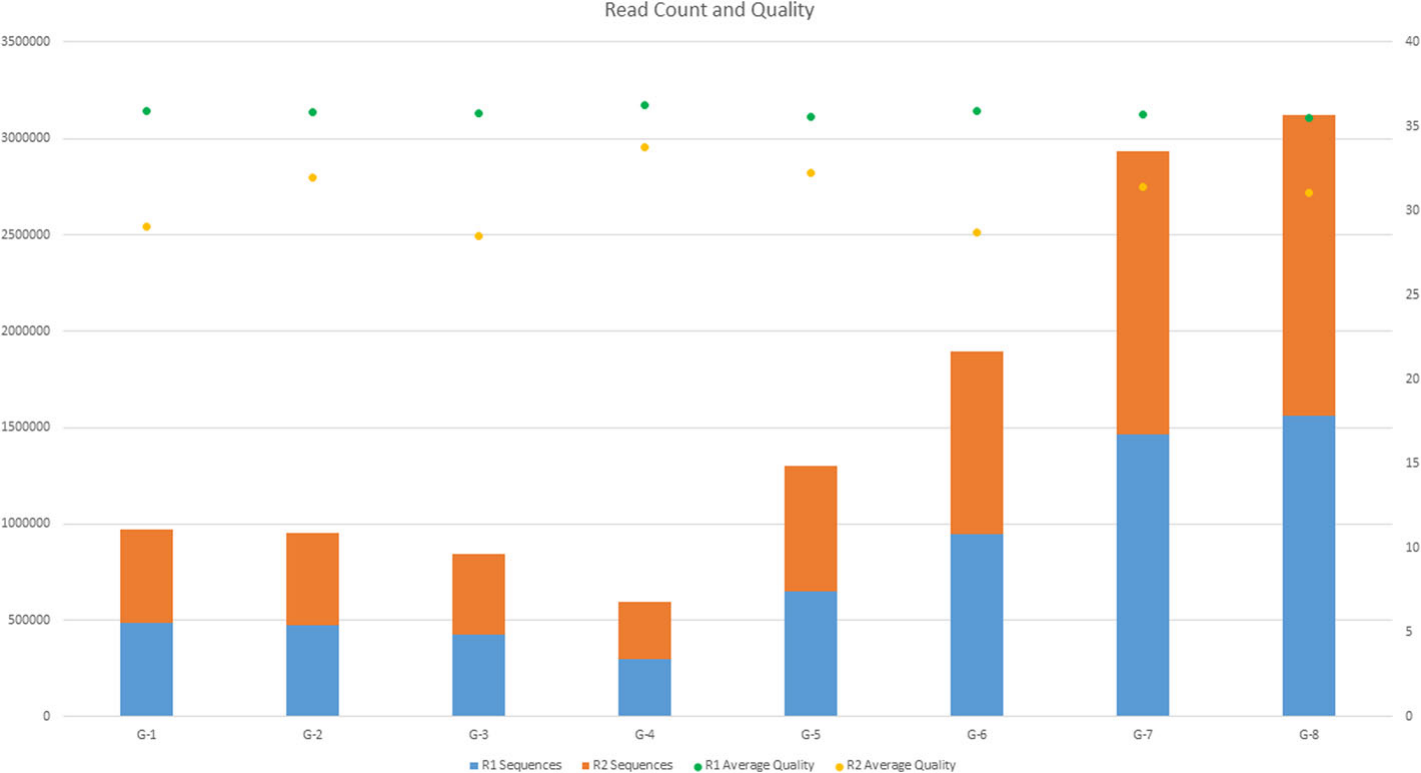
Read count and quality statistics for samples used in runtime testing. Left-side Y-axis represents the number of reads. Right-side Y-axis represents PHRED quality score average

**Fig. 5:**
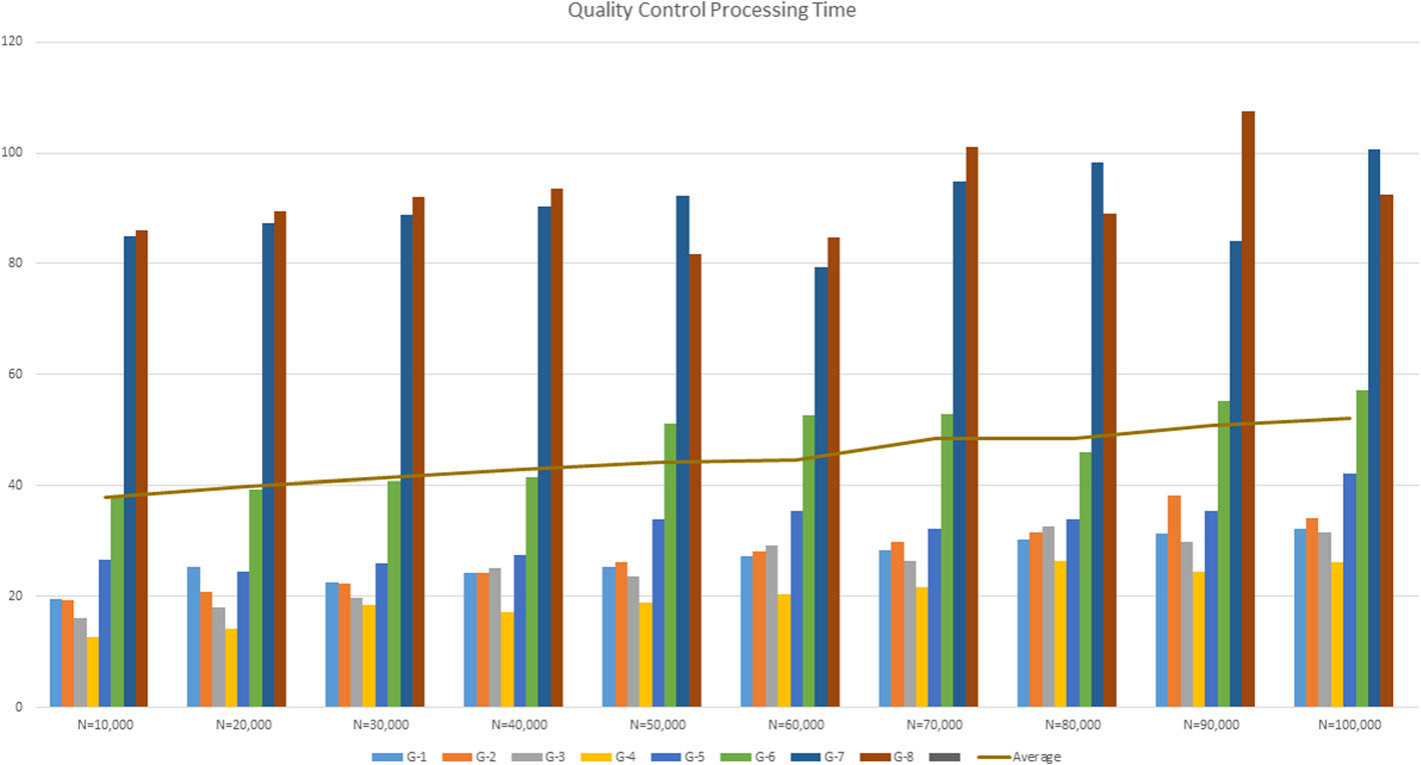
Runtime measurements for Quality Control tasks spanning subsampling levels of 10,000–100,000 read pairs. Y-axis unit in minutes

**Fig. 6:**
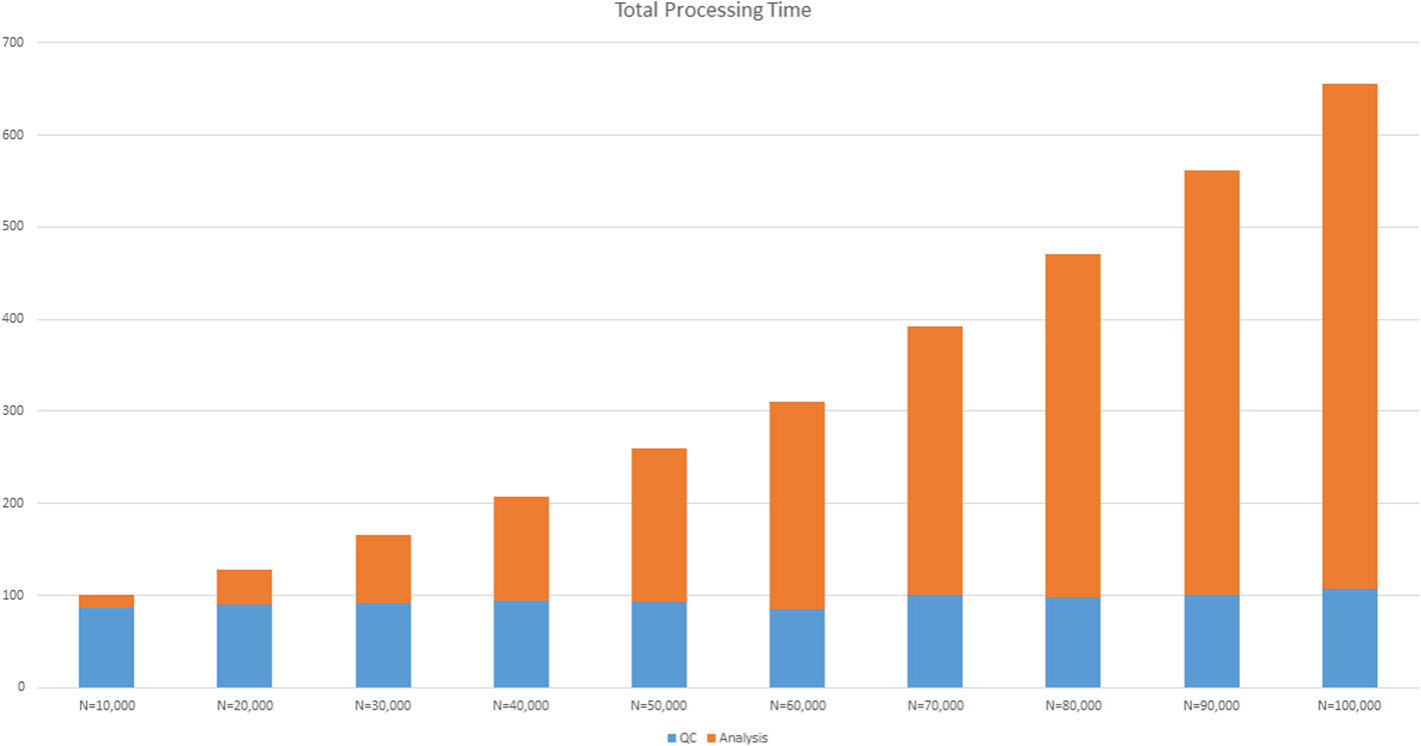
Total processing time for all samples of the specified subsampling level to complete. Y-axis in minutes

**Fig. 7:**
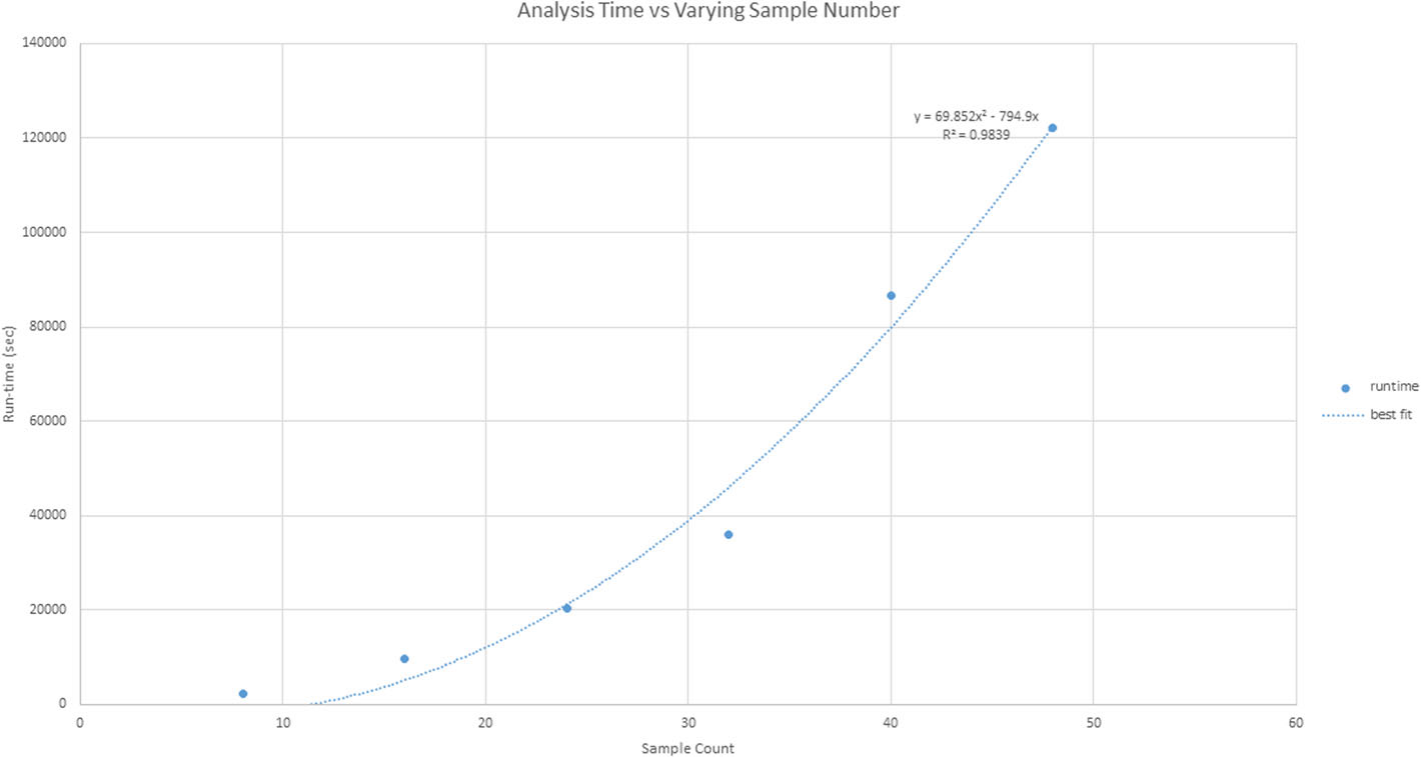
The all-pairs minimum distance calculation portion of the Analysis task time with varying number of input samples. Best fit line calculated using the quadratic least squares regression

Runtime analysis was performed on the GHOST Quality Assurance (QA) platform, which is identical to that of the production instance except for the number of nodes in the EC2 configuration, having 10 nodes (2 management, eight compute). Eight single-subtyped unrelated samples in which no pairwise grouping triggers the metric filter were chosen across a range of file sizes and used to conduct speed testing on the QA tier GHOST instance (Fig. 4). The QC task was tested against a range of subsampling levels to determine its effect on the runtime (Fig. 5). Runtimes were assessed for all samples submitted with respect to the total QC task execution time, the all-pairs minimum distance calculation portion of the Analysis task time, and the combined time for both (Fig. 6). The QC task total execution time remained relatively stable with respect to subsampling level exhibiting a linear increase with small slope. The all-pairs minimum distance calculation execution time retained a linear characteristic with a more aggressive slope across the subsampling range tested. However, the pairs generated by n samples is n(n-1)/2, and this can be observed in the runtime when varying the sample number while holding the number of nodes and subsampling level constant (Fig. 7). With the current production configuration, subsampling at the level of 20,000 read pairs, GHOST can process an entire MiSeq run in approximately 3 h.

### Reproducibility

**Fig. 8:**
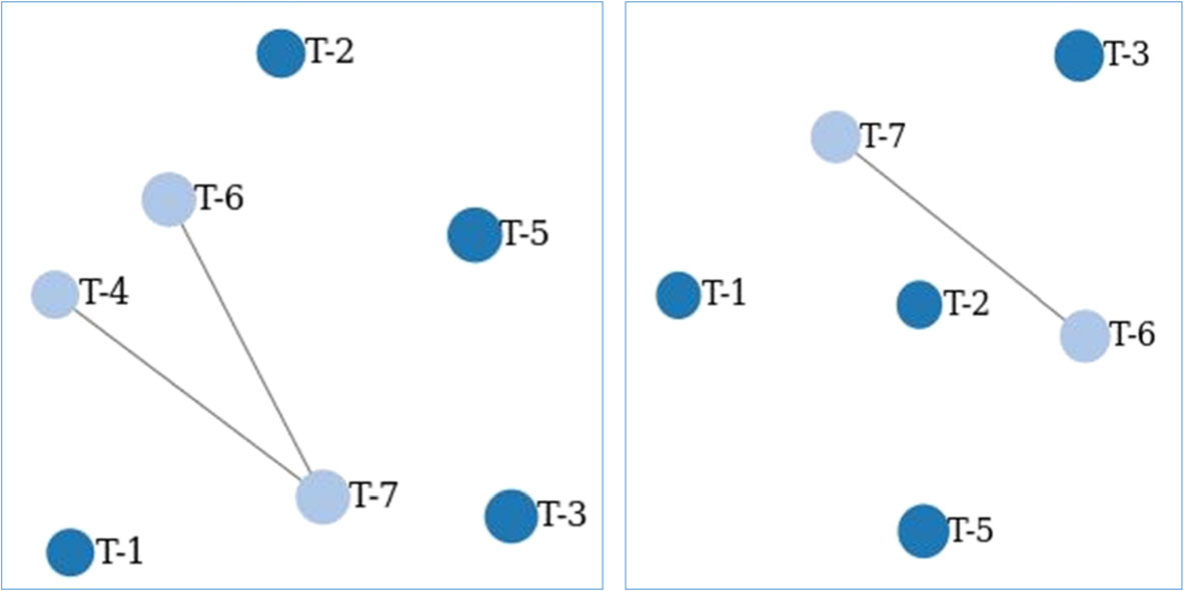
Dark blue balls represent unrelated samples. Light blue balls represent samples in a cluster. Lines represent relatedness. Left shows GHOST linkage results for 5 of the 6 libraries constructed during the state health department GHOST Training in November, 2015. Right shows Library 5 GHOST linkage results, which showed the absence of T-4 due to loss of pellet during library preparation

A series of libraries with identical composition were used to evaluate consistency of GHOST results. Six libraries were prepared from six commercially available serum specimens, with each specimen containing a single known HCV subtype. In each library, five samples were prepared using a single specimen (1a, 1b, 2 k, 3a, and 6f), two samples were prepared using a combination of specimens, one mixture of the specimens containing HCV subtypes 3a and 4a, one mixture of 2a and 4a, and a negative control (Table 1). The libraries were divided into two sets of three, and each set was sequenced using a different MiSeq instrument. GHOST results from each of the six libraries were consistent in linkage (Fig. 8, left side), except for library five which, owing to loss of pellet during library preparation did not contain a product for one sample consisting of a combined serum mixture. Similar to the negative controls, this sample had an unusually low yield of reads, did not pass GHOST’s secondary identifier filter, and was automatically removed from further analysis (Fig. 8, right side).

**Table 1:** Serum sources used to construct MiSeq libraries

Name	Source	Subtype
T-1	Single	1a
T-2	Single	1b
T-3	Single	2 k
T-4	Single	3a
T-5	Single	6f
T-6	Combination	2b/4a
T-7	Combination	3a/4a
T-8	none	none

### Specificity

**Fig. 9:**
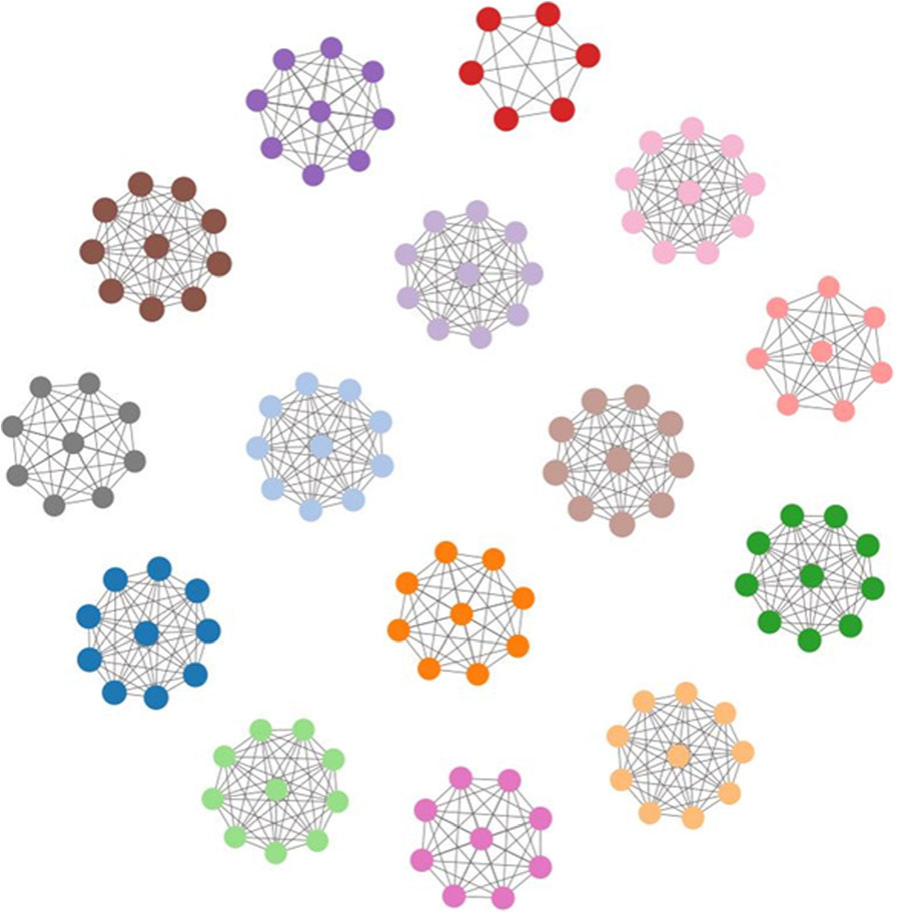
GHOST output for ten-fold subsampling (*N* = 20,000) of 16 samples with no epidemiological evidence of intra-group transmission. Fifteen clicks are present, as one sample did not have sufficient product for sequencing, and subsequent sequence subsets did not pass GHOST preprocessing filters

To observe the rate of false linkages, sixteen epidemiologically unrelated HCV samples were sequenced using two MiSeq runs in groups of eight (Unrelated Collection in Table 2). One sample did not appear to have a product at the end of library construction, and sequence yield supported this observation. The remaining sample files were then randomly sampled (*N* = 20,000) 10 times, and submitted to GHOST to obtain the transmission network. As expected, GHOST analysis produced linkages only between subsamples from the same individual (Fig. 9). In addition, no linkages were observed between subsamples of different origin, nor was there any linkage evident from intra-run read mis-assignment.

**Table 2:** Summary table of data used in the study

Collection	Classification	Samples number	Origin
G1–8	Unrelated	8	CDC Archive
T1–8	Unrelated	8	Artificial
Unrelated Collection	Unrelated	16	CDC Archive
Transmission Collection	Related	8	Outbreak
Time Series Collection	Related	8	CDC Archive
Spike Collection	Related	8	Artificial

### Sensitivity

The following test was devised to observe the recovery rate of expected linkages with a varying level of random sampling. Three libraries were created from sample collections with various types of shared populations to survey GHOST’s link detection sensitivity in these shared population types at a progressively declining random sampling level. The collections included 8 samples containing 3 pairs with accompanying epidemiological evidence supporting intra-pair transmission (Transmission Collection in Table 2), 8 samples collected from four HCV-infected individuals in time series pairs with varying time intervals between collection points (Time Series Collection in Table 2), and 8 samples including 3 spiked serum mixtures at 10%, 1%, and 1% mixture levels (Spike Collection in Table 2). These 3 libraries were sequenced using 3 separate MiSeq runs and submitted to GHOST with an exponentially decreasing subsampling parameter (*N* = 10^4^–10^1^). Both the Transmission and Time Series Collections persisted in maintaining the expected linkage from N = 10^4^ to *N* = 10^2^, but only retained ~27% and 35% of the expected links at the *N* = 10^1^ level, However, they both retained 100% expected linkage with the minimum frequency filter relaxed to 1 from the default of 2. The Spike Collection showed a more gradual nature in the decline of links formed as the random sampling level decreased. For all collections, there were no instances of unexpected links observed (Table 3).

**Table 3:** GHOST accuracy at subsampling levels *N* = 10^4^ to *N* = 10^1^. The final column shows linkage percent when the filter requirement for the minimum frequency of a unique sequence to create linkage is reduced from the default of 2 to 1

Dataset	Links expected	*N* = 10,000	*N* = 1000	*N* = 100	*N* = 10 (*m* = 2)	*N* = 10 (*m* = 1)
Transmission	30	100.00%	100.00%	100.00%	26.67%	100.00%
Time Series	40	100.00%	100.00%	100.00%	35.00%	100.00%
Spike	40	100.00%	75.00%	27.50%	0.00%	57.50%

## Discussion

### Transmission detection

GHOST allows the accurate and cost-effective detection of possible HCV transmission clusters with high reproducibility, sensitivity and specificity. Traditionally, phylogenetic reconstructions are used to determine ancestral relationships. The GHOST’s core pipeline works by calculating genetic distances and reporting a link where the distance is below an experimentally validated threshold [23]. This threshold method is computationally efficient and allows for a graphical representation of the expected transmission network that is intuitive for users.

GHOST analysis is intended to be easy to execute with output results that are easy to interpret, but it is important to state the limits on what can be inferred. In a real-life scenario with HCV-infected individuals whose infections are derived from a common source population, there are multiple possible chains of events that could explain the causality of occurrence. It is not always known whether a common source was sampled during outbreak investigations. Furthermore, there could be any number of individuals not included in the cohort who are points in the transmission chain between individuals in the study and their common source. In its current state, GHOST cannot be used to make assertions of source identification or the directionality of transmission. GHOST is intended to be used as a tool for the detection of transmission clusters, and while GHOST analysis may aid and support a particular hypothesis, traditional epidemiological investigations into such claims remain necessary.

GHOST’s current distance threshold (0.037) developed using End-Point Limiting-Dilution (EPLD) data and validated on the 454 platform (Life Sciences, Roche) data [23] was applied here to Illumina data, which are usually more abundant. It was shown that an increase in the read sample size results in a greater probability of identifying shared or genetically close intra-host HCV variants in specimens obtained from epidemiologically defined transmission pairs without affecting genetic relatedness among unrelated samples [23], thus improving reliability of detection of transmission links. Hence, the GHOST-based analysis of the Illumina data using the established threshold provides a more reliable estimate of transmission clusters than analysis of sequences generated using EPLD and 454 technologies. Recently, new methods have been developed that make use of sample-specific differences [22, 32]. Application of advanced clustering techniques and probabilistic evolutionary models in conjunction with the implemented GHOST workflows should further improve reliability of transmission detection.

### Genotyping

Genotyping information is provided to characterize HCV sequences found within a sample to the sub-type level. It should be noted that GHOST uses HVR1 in calculating the sample linkage because the high rate of evolution is conducive to determination of recent transmission events. However, this rate of evolution in conjunction with the region’s small size are not conducive to accurate genotyping at the subtype level. The subtype assignment is used in GHOST only to cluster sequences in QC tasks. This clustering was not intended for accurate assessment of subtypes, which have little bearing on the detection of transmission links. Although care was taken to make genotyping assertions as consistent and accurate as possible, sequence assignments to some rare subtypes using HVR1 employed in GHOST may hypothetically differ from assignment using other HCV genomic regions and, if important, should be supported by supplemental evaluation.

### Experimental considerations

Early efforts in the transition of GHOST from 454Jr-based to MiSeq-based were challenging due to GHOST’s high level of sensitivity in detection of minority variants combined with a broad spectrum of multiplexing errors inherent to the platform. The introduction of a second set of identifiers in the library construction protocol was essential but not entirely sufficient to eliminate all falsely assigned reads. The restriction requiring a minimum frequency of 2 for any sequence to participate in linkage, combined with the restriction requiring a 25% maximum of valid but non-majority secondary identifiers detected per sample has thus far shown reliable in eliminating false positive links. However, these two restrictions may be adjusted as warranted by validating with data as the GHOST pilot progresses.

As with any experimental procedure, GHOST can be greatly affected by the quality of input data going into the analysis. The GHOST software is designed to produce accurate results despite various common types of NGS sequence irregularities. However, laboratory-related contamination and other types of quality control issues can produce erroneous results. Not all of these laboratory-based issues can be resolved using software alone. Nevertheless, application of automated laboratory equipment and robotic workstations, besides reducing human errors, offers automatic availability of additional data associated with processing of each tested serum specimen in each laboratory procedure, providing opportunity to develop novel quality control models for tracking potential laboratory artifacts affecting accuracy of transmission detection.

### Modularity

Although GHOST’s current analysis is for the detection of HCV transmission, the modular nature of GHOST potentially allows for other analytical modules using the same amplicon data. For instance, it has been shown that intra-host variability is correlated with the duration of the infection [33–35], which can be used to infer directionality in transmission events and distinguish between acute and chronic cases. Similarly, GHOST was originally designed to target HVR1, but with the redesign of GHOST to accommodate the Illumina-based platform, the system was rebuilt to be target-agnostic. It is currently being adapted to allow usage of additional HCV genomic regions for outbreak detection and diagnostics. This will be crucial for both supplementary support and when alternative targets are needed in lieu of a failure to sequence the primary target. GHOST is not HCV-specific. The modular GHOST infrastructure is accommodative of any model, including models for other hepatitis viruses or any other pathogens. Currently, we are exploring the application of this system in a diversity of different pathogen-specific applications.

## Conclusions

GHOST software described here is a novel diagnostic system that hosts and operates a set of computational models. The GHOST models act as virtual diagnostic assays, which use NGS data the same way as laboratory-based serological and molecular assays use serum specimens. GHOST has been fully adapted to the Illumina platform and deployed to a cloud environment. Currently, it is in a pilot phase, being evaluated in several public health laboratories. Detection of transmission networks in real-time during outbreak investigation and surveillance activities are crucial for implementation of timely public health interventions to interrupt transmissions. Molecular epidemiological investigation is very complex and requires experience in molecular technologies, epidemiology and computational analysis. GHOST is a web-based technology that allows for automatic extraction of public health relevant information from NGS data, enabling all end users, independent of their level of expertise, to analyze and visualize expected transmission clusters in a cost-effective, standardized and real-time way for supporting outbreak investigation and molecular surveillance. Although GHOST was originally developed for hepatitis C surveillance, its modular structure is readily applicable to other infectious diseases.

## Abbreviations

AWS: Amazon Web Services
CDC: Centers for Disease Control and Prevention
DRMAA: Distributed Resource Management Application API
EC2: Elastic Compute Cloud
EPLD: End-Point Limiting Dilution
GHOST: Global Hepatitis Outbreak and Surveillance Technology
HBV: Hepatitis B virus
HCC: Hepatocellular carcinoma
HCV: Hepatitis C Virus
HIV: Human immunodeficiency virus
HMM: Hidden Markov Model
IDU: Injection drug use
MSA: Multiple sequence alignment
NGS: Next Generation Sequencing
QA: Quality assurance
QC: Quality Control
S3: Simple Storage Service
UDS: Ultra-Deep Sequencing
